# Finding what works: identification of implementation strategies for the integration of methadone maintenance therapy and HIV services in Vietnam

**DOI:** 10.1186/s13012-016-0420-8

**Published:** 2016-04-20

**Authors:** Vivian F. Go, Giuliana J. Morales, Nguyen Tuyet Mai, Ross C. Brownson, Tran Viet Ha, William C. Miller

**Affiliations:** 1 Department of Health Behavior, University of North Carolina Gillings School of Global Public Health, Chapel Hill, NC USA; 2 Prevention Research Center in St. Louis, Brown School, Washington University, St. Louis, MO USA; 3 Division of Public Health Sciences and Alvin J. Siteman Cancer Center, School of Medicine, Washington University, St. Louis, MO USA; 4 Department of Epidemiology, University of North Carolina Gillings School of Global Public Health, Chapel Hill, NC USA; 5 Department of Medicine, School of Medicine, University of North Carolina at Chapel Hill, Chapel Hill, NC USA; 6 Current affiliation: Division of Epidemiology, College of Public Health, The Ohio State University, Columbus, OH USA

**Keywords:** Vietnam, MMT/HIV integration, People who inject drugs, Implementation strategies, Implementation research, Implementation science

## Abstract

**Background:**

Integration of methadone maintenance therapy (MMT) and HIV services is an evidence-based intervention (EBI) that benefits HIV care and reduces costs. While MMT/HIV integration is recommended by the World Health Organization and the Centers for Disease Control and Prevention, it is not widely implemented, due to organizational and operational barriers. Our study applied an innovative process to identify implementation strategies to address these barriers.

**Methods:**

Our process was adapted from the Expert Recommendations for Implementing Change (ERIC) protocol and consisted of two main phases. In Phase 1, we conducted 16 in-depth interviews with stakeholders and developed matrices to display barriers to integration. In Phase 2, we selected implementation strategies that addressed the barriers identified in Phase 1 and conducted a poll to vote on the most important and feasible strategies among a panel with expertise in cultural context and implementation science.

**Results:**

Barriers fell into two broad categories: policy and programmatic. At the policy level, barriers included lack of a national mandate, different structures (MMT vs. HIV clinic) for cost reimbursement and staff salaries, and resistance on the part of staff to take on additional tasks without compensation. Programmatic barriers included the need for cross-training in MMT and HIV tasks, staff accountability, and commitment from local leaders. In Phase 2, we focused on programmatic challenges. Based on voting results and iterative dialogue with our expert panel, we selected several implementation strategies in the domains of technical assistance, staff accountability, and local commitment that targeted these barriers.

**Conclusions:**

Key programmatic barriers to MMT/HIV integration in Vietnam may be addressed through implementation strategies that focus on technical assistance, staff accountability, and local commitment. Our process of identifying implementation strategies was simple, low cost, and potentially replicable to other settings.

**Electronic supplementary material:**

The online version of this article (doi:10.1186/s13012-016-0420-8) contains supplementary material, which is available to authorized users.

## Background

The process of translating evidence-based interventions (EBIs) to real-world settings is a critical step in the research to practice continuum. But this step is frequently neglected in the public health sector [1–4]. The field of implementation science has rapidly expanded to address this gap within public health [5] and, more specifically, within HIV research [6–8]. While the effectiveness of EBIs can be achieved in a real-world HIV context [9–11], *how* EBIs are implemented clearly affects their effectiveness and sustainability [12]. In other words, implementation outcomes, such as feasibility and acceptability, are directly linked to HIV effectiveness outcomes in the real world [5].

Several evidence-based implementation strategies are being used to facilitate the complex process of moving EBIs into real-world settings [2, 4, 13, 14]. Although many implementation strategies [15, 16] and taxonomies [16–18] have been developed, the potential effectiveness of these implementation strategies in specific settings is uncertain [4, 14]. Service settings vary substantially with regard to contextual factors such as patient characteristics [19, 20], provider attitudes toward an EBI [21], organizational readiness [22], leadership [23], and policies [24]. These contextual factors can, in turn, influence the success of implementation strategies [4, 14]. While strategies designed to address context-specific barriers and facilitators are more likely to succeed [4, 14], the process of selecting the most effective strategy for an EBI in a given context can be overwhelming. Recently, guidelines to facilitate this process of selecting implementation strategies for specific contexts have been developed [14]. For example, the Expert Recommendations for Implementing Change (ERIC) protocol is a four-stage, mixed-methods process to develop expert recommendations regarding implementation strategies for a given context [14].

Integration of opioid substitution therapy, such as methadone maintenance therapy (MMT), and HIV related services is an EBI that leads to increased engagement of people who inject drugs (PWID) in HIV prevention and treatment [25–33]. HIV-infected PWID who participate in integrated drug treatment and HIV services experience several benefits [34]. They experience less bureaucracy accessing and engaging in services; increased monitoring of drug interactions and side effects from MMT and the provision of antiretroviral treatment (ART) for HIV [35–45]; reduced HIV stigma from providers; consolidated drug use and HIV counseling; and health care delivery systems that are targeted and user-friendly [46–48]. In addition, integration of drug treatment and HIV care services reduces costs [49, 50] and is cost-effective [51, 52], which is critically important in low- and middle-income countries. Combining services increases efficiency and lowers costs by reducing space requirements and building a cadre of providers with multiple skillsets.

Opioid substitution/HIV care integration is endorsed by international agencies [53, 54] to address disproportionately low access to ART among HIV-infected PWID [55]. Only one in ten PWID living with HIV receives ART [56]. Those who have accessed ART have generally low adherence and poor HIV disease-related outcomes [57–59]. When PWID living with HIV have access to MMT, they increase ART uptake [60–62] and ART adherence [58, 60, 63–69], have improved HIV viral suppression [68–70], and live longer [71]. Additionally, integrating MMT and HIV services optimizes these effects by increasing ART initiation and adherence [25–27] and improving viral suppression [26, 27].

Despite improved outcomes for PWID with MMT/HIV service integration, the most effective approaches to the implementation of MMT/HIV integration have not been identified [72], and integrated services have not been implemented widely [73, 74]. Past integration efforts have faced multiple barriers including organizational and political constraints, overburdened clinicians struggling to take on new responsibilities, and a lack of leadership investment at all levels [50, 75–77]. In Vietnam, integrated MMT/HIV clinics, piloted by the Vietnam Administration for HIV/AIDS Control (VAAC), the division of the Ministry of Health responsible for HIV services, have had varied success [78, 79], underscoring the need for implementation strategies to ensure effective implementation across integrated clinics. In this study, we sought to understand common barriers and subsequently select implementation strategies that could effectively address core challenges for integrated MMT/HIV clinics in Vietnam. We applied an innovative implementation science approach based on the ERIC protocol, to assess barriers and facilitators to MMT/HIV integration and used this assessment as a foundation for identifying appropriate implementation strategies within the Vietnam context.

## Methods

To identify implementation strategies, we used a streamlined process adapted from the ERIC protocol by Waltz et al. although we omitted the Delphi process and concept mapping given limited resources and time [14]. We used the ERIC methodological template to advance our two core aims: (1) understanding barriers and facilitators to MMT/HIV integration using qualitative interviews and a process of prioritization with multiple stakeholders and (2) identifying the most important and feasible implementation strategies for effective MMT/HIV integration through voting and an iterative dialogue with our expert panel.

### Phase 1

We conducted semi-structured in-depth interviews (IDIs) with key stakeholders in Vietnam between December 2014 and February 2015 to explore the range of definitions and attitudes regarding MMT/HIV integration as well as barriers and facilitators to the process. The study was reviewed and approved by the University of North Carolina at Chapel Hill (UNC) Institutional Review Board and the Hanoi School of Public Health Institutional Review Board.

Participants (*n* = 16) were purposively selected based on their role in the Vietnam health system and their experience with integrated MMT/HIV clinics. They represented three categories: (1) c*entral*-*level stakeholders*: VAAC staff (*n* = 3) and a staff member from a non-governmental organization (NGO) that provides technical assistance to MMT/HIV pilot integration clinics throughout Vietnam (*n* = 1); (2) *Department of Health and clinic directors* (*n* = 5): and (3) *clinic staff*: care providers (*n* = 7) from stand-alone clinics in one province and pilot integration clinics in two provinces. Interview guide content was based on our literature review of MMT/HIV integration and informal meetings with NGO staff members with experience in integration in Vietnam. Separate guides were developed for each participant category (Additional files 1, 2, 3, 4, 5). Content was similar across guides but tailored to probe on specific experiences and glean insights from each participant category. For example, central-level stakeholders were asked, “Let’s say that you wanted to initiate a national goal to integrate HIV and MMT services in Vietnam for the VAAC. Walk me through what exactly would need to be done in order to integrate services from the national level, down to the clinic level” whereas clinic staff level were asked, “Can you tell me how (you think) integration of services [would] affect you and your role?”

IDIs were conducted by experienced ethnographers who participated in an intensive 1-week training on the objectives of the study and the interview guides. The interviews were conducted privately in participants’ offices or at the clinics for about 1 hour. Interviews were audiotaped, transcribed, and translated from Vietnamese to English. Permanent UNC Vietnamese staff, who are fluent in English and who have more than 5 years of experience in technical translations for our research team, conducted the translations. The codebook was developed based on the interview guides and an initial review of the translated interviews. Codes included definitions, attitudes, barriers, facilitators, and outcomes of MMT/HIV integration. Translated interviews were coded and reports were generated for analysis using Atlas.ti v.7 data analysis software. Ten percent of transcripts were double-coded by two coders to ensure coding reliability. Any inconsistencies were discussed and resolved. The analysis consisted of a search for themes, trends, and emerging patterns among the three stakeholder categories: (1) central-level stakeholders, (2) Department of Health and clinic directors, and (3) clinic staff. Based on coded and categorized data, we created a comprehensive matrix to identify common threads and contrasts across and within stakeholder levels. For example, stakeholders at all levels identified ongoing technical assistance as an important facilitator while only clinic directors and staff voiced concerns about low staff morale due to the addition of new tasks to existing duties.

We reviewed the matrix and divided barriers and facilitators into two categories: policy and programmatic. We confirmed findings at this stage through a separate face-to-face meeting with each of the central-level stakeholders in Vietnam, with bi-directional translation provided by a Vietnamese member of our UNC research team as needed. Policy barriers comprised inconsistencies in service structures and staffing in MMT and HIV clinics. Programmatic challenges comprised technical assistance for clinic staff, staff accountability, and local commitment. In Phase 2, we focused on implementation strategies to address programmatic challenges since they were potentially modifiable through external intervention.

### Phase 2

Using a compilation of 73 implementation strategies [16], we identified 20 specific implementation strategies that aligned with the three domains of programmatic barriers and facilitators identified in Phase 1. For example, under “Training,” we listed Distribute Educational Materials; Use Train-the Trainer Strategies; Conduct Educational Outreach Visits; Conduct Ongoing Training; Develop Educational Materials; Make Training Dynamic; Provide Local Technical Assistance; and Provide Ongoing Consultation. A challenge was to create a list that was comprehensive, concise, and where strategies were discrete. Working with two experts from the Implementation Research Institute in Washington University in St. Louis [80] and the Gillings School of Global Public Health at UNC, we circulated our preliminary list, identified overlapping strategies, and, where appropriate, combined complementary strategies. For example, we determined that the strategy, “Provide Ongoing Consultation” overlapped with the strategy “Provide Local Technical Assistance” and so created a combined strategy: “External Technical Assistance and Ongoing Consultation.” By combining strategies where appropriate, we were able to reduce the total number of potential implementation strategies to 16 within the three programmatic domains.

To select the most appropriate implementation strategies from the 16, we asked our panel to vote using a web-based, self-administered survey. The panel comprised four Vietnamese stakeholders who were familiar with the Vietnamese context of MMT/HIV integration and five implementation science experts who were familiar with the application of implementation strategies in a range of settings. Implementation science experts were selected on the basis of their implementation science-related publications, mentorship, and grant records (i.e., participation in at least one of each). The size of our expert panel (*n* = 9) was determined by balancing considerations of practicality with the ability to capture a range of perspectives. The survey briefly introduced MMT/HIV integration, the EBI, and the Vietnam context. Our expert panel scored each potential strategy from 1 to 10 in terms of feasibility and importance, 1 being the least feasible and important and 10 being the most feasible and important. We asked members to consider feasibility and importance jointly in their score. Members were able to weigh the relative significance of feasibility and importance into their overall recommendation score, as they felt appropriate. The survey also included space to comment on scoring of the technical assistance, staff accountability and local commitment sections. These qualitative data allowed us to characterize variability around the recommendations and recognize the importance of cultural specificity of implementation strategies. The median, mean, and range of scores of each strategy, in combination with the open-ended comments, determined the final selection of implementation strategies.

## Results

First, we present Phase 1 findings that include definitions of integration, attitudes toward integration, and barriers and facilitators to integration. We then present Phase 2 findings, which describe the identification of implementation strategies.

### Phase 1

#### Definitions of integration

Not all stakeholders had direct experience with MMT/HIV integration, yet all knew about integration and identified co-location of services, shared clinic staff, and joint management as essential components of integration. Clinic providers from a pilot integration clinic emphasized the need for all three components, noting that their clinic has co-located MMT and HIV departments but is not fully integrated since the departments do not share a management board or staff. In addition to these three components, central-level stakeholders suggested integrated records as an important dimension of integration.

Three approaches to the integration of clinics were identified, (1) building an MMT/HIV clinic from the start if neither MMT nor HIV clinic already exists, (2) adding MMT services to an existing HIV clinic using existing facilities and staff, and (3) combining two or more existing MMT and HIV clinics by reducing facilities and staffing structures. The third model was considered the most challenging model to implement by central-level stakeholders.

#### Attitudes toward integration

Overall, participants were receptive to service integration, noting the benefits to both patients and the health system. The most important benefit was identified as better access to services and care for MMT and HIV patients. Specifically, integration was seen as reducing transportation, financial, and logistical barriers to accessing and engaging in MMT/HIV services. At the clinic level, integration was viewed as enabling staff to provide better care and treatment for patients. In integrated clinics, staff can monitor treatment progress and ensure patients receive appropriate counseling and dosages. One clinic director’s experience illustrates the value of integration from the perspective of a stand-alone clinic:

When we implement, there are many advantages, the biggest advantage is for the patients…they do not have to go anywhere, the methadone treatment doctors are also the ones who treat HIV, they understand both methadone, ARV [antiretroviral] for appropriate treatment for patients. They monitor their patients daily, then they can extract early and timely all the side effects as well as the challenges, obstacles to the patients… I think that whatever is beneficiary for the community, useful for the patients, we should do. (Clinic Director, Stand-alone Clinic)

Central-level stakeholders and some clinic directors and providers also noted that integration could save resources by combining or reducing staff and space. The cost-effectiveness of integration is particularly important for MMT and HIV programs, which have been supported almost exclusively by external funders, and which are now facing imminent withdrawal of external funding. Stakeholders at the central and clinic director levels identified the need to transfer program support to the VAAC. Clinic directors in particular explained that government funds would need to replace external funds to ensure the successful implementation and sustainability of MMT and/or HIV services. Central-level stakeholders reiterated this point: “Integration is the solution [to international withdrawal of funds]. Integration is a form of personnel and cost saving for both government and patients.” (VAAC)

#### Barriers and facilitators to integration

Although a range of barriers and facilitators to integration were described during the interviews, most stakeholders referenced five main domains. Two of these are modifiable at the policy level (service and staffing structures) and three are modifiable at the programmatic level (technical assistance for clinic staff, staff accountability, and local commitment). Domains that were mentioned by the simple majority of participants are included in Tables 1 and 2.

**Table 1:** Barriers and facilitators that are modifiable at the policy level

Level	Domain	Barriers	Facilitators
Central	Service structures	• Inconsistent requirements and procedures between MMT and HIV services (staffing, renumeration, medical dispensing, health insurance) • Multiple management units (district vs. province) • Separate patient record systems	• Step-by-step instructions for implementation at the district and province levels • Revised medical dispensing schedule • Unified patient record system
	Staffing structures	• None	• Transition of staff from program status to government employee status
DOH and clinic directors	Service structures	• Lack of legal framework for integration • Inconsistent requirements and procedures between MMT and HIV services (common prices, health insurance, certification) • Multiple management units	• Legal framework and regulations for providing integrated MMT and HIV services • Provision of health insurance for both MMT and HIV services
	Staffing structures	• Lack of full-time, permanent staff and inability to attract external human resources • Lack of renumeration that is commensurate to increased responsibilities • High-risk working environment	• None
Clinic providers	Service structures	• Inconsistent requirements and procedures between MMT and HIV services (staffing, renumeration, medical dispensing, health insurance) • Lack of unified reporting system	• None
	Staffing structures	• Lack of renumeration that is commensurate to increased responsibilities (high workload) • Lack of staff and inability to attract external human resources • Lack of government staff positions and program staff treated differently • High-risk working environment	• None

**Table 2:** Barriers and facilitators that are modifiable at the programmatic level

Level	Domain	Barriers	Facilitators
Central	Technical assistance	• None reported	• Human resource training and certification • Technical assistance to facilitate process
	Accountability	• None reported	• Renumeration for monitoring and evaluation
	Local commitment	• None reported	• Local leadership buy-in informed by project evidence
DOH and clinic Directors	Technical assistance	• Lack of human resource training • Lack of integration model description • Patient discomfort sharing facilities with IDUs	• Human resource training and certification
	Accountability	• Lack of monitoring and evaluation reporting criteria	• Support from medical director and other medical departments • Social support network for clinic staff
	Local commitment	• Lack of province-to-province learning opportunities	• None reported
Clinic providers	Technical assistance	• Limited knowledge of integrated services • Lengthy time lapse between training and clinic start-up • Disruptive IDU patient or drug seller behavior	• Human resource training and practice • Educational materials • Coaching support • Proactive information sharing between departments
	Accountability	• None reported	• Knowledge of clinic staff responsibilities • Proactive information sharing between departments • Collaborative work environment
	Local commitment	• Lack of clinic staff buy-in	• Clinic engagement with community

### Modifiable at the policy level

#### Service structures

All clinic directors cited the need for a central-level decree or legal framework to successfully implement integration. They noted that official guidance would be helpful for integrated MMT/HIV services due to the inconsistent requirements and procedures between the two services and the sensitive nature of substance abuse and HIV treatment.

While current systems for medical records, administrative procedures, forms, and bookkeeping in MMT and HIV clinics have been established separately, many expressed the belief that one integrated system would be more effective. Clinic providers noted that an integrated record system would provide complete patient records and would enable them to monitor drug interactions and provide appropriate care and treatment. In addition, staffing and medical dispensing guidelines between MMT and HIV services are currently inconsistent. For example, methadone doses are dispensed daily while ARV doses are dispensed monthly. In MMT clinics, staff are often required to work on weekends and holidays. A few stakeholders provided suggestions to overcome these barriers, such as permitting clinic staff to administer methadone doses for weekends and holidays in advance.

In addition to intra-service operational inconsistencies, differences in client fees and provider salaries are challenges to integration. Specifically, HIV services currently are covered by health insurance but MMT services are not. Furthermore, MMT providers receive a “hardship” allowance that HIV providers do not receive. Central-level stakeholders recognized these differences and the need to align fee and salary differences between services.

#### Staffing

Clinic directors and providers frequently cited heavy workloads and staff shortages as challenges to integration. While the need to invest in additional staff was widely recognized at the clinic level, the lack of permanent positions, low salaries, high-risk working environments, and stigma against PWID and HIV patients were barriers to recruiting additional staff.

Clinic directors in integration pilot clinics viewed training providers on new tasks as an alternative to recruiting additional staff. However, most clinic providers in the integrated clinics reported feeling tired and overwhelmed at the prospect of additional tasks being added to their existing responsibilities. In several cases, clinic providers expressed dissatisfaction with the idea of taking on additional responsibilities with no additional salary, noting that their salary should be commensurate with their responsibilities. This disparity between increased responsibilities and static salary has decreased staff morale in both successful and unsuccessful integration pilot clinics.

Clinic directors recognized the potential (or reality) of decreased staff morale and discussed strategies that have successfully facilitated a collaborative, supportive environment to increase staff motivation. These strategies are discussed in further detail below under “staff accountability.” At the policy level, central-level stakeholders are working on addressing issues such as task shifting and staffing reduction.

### Modifiable at the programmatic level

#### Technical assistance for clinic staff

Inadequate staff training was the most commonly perceived barrier to integration at the programmatic level. Both clinic directors and providers reported lack of skills for the provision of integrated services as a challenge. At all levels, staff training and certification were seen as simple but important facilitators to successful integration.

Clinic providers in stand-alone clinics perceived that learning how to provide integrated services would not be difficult. Additionally, clinic providers in both integration pilot clinics and stand-alone clinics suggested practice-based learning as an effective teaching approach. A clinic provider recommended decreasing the lag time between training and integration uptake to ensure that clinic staff are able to apply their training immediately.

Outside of staff training, clinic providers also highlighted technical assistance activities, such as receiving educational materials and coaching assistance as needed, to facilitate the integration process.

#### Staff accountability

At the clinic level, clinic directors and providers from integration pilot clinics explained that increasing staff accountability would also facilitate integration implementation. Staff accountability can be increased through knowledge and information sharing as well as collaboration and social support among clinic staff.

For knowledge and information sharing in an integrated clinic, clinic-level stakeholders highlighted the importance of clarifying staff responsibilities ranging from bookkeeping and counseling to testing and reporting. One clinic provider also mentioned that although information is shared during meetings, information sharing and internal communications should also be proactive and in real time. Additionally, a clinic director suggested implementing reporting criteria, noting that it is essential to monitor and evaluate integrated services at the clinic level:

…from [the] beginning, we haven’t got description on this model in our hospital as well as reporting criteria, thus we don’t know where and how to report MMT results. Our efforts cannot not be measured and evaluated. (Clinic Director, Integration Pilot Clinic 1)

For collaboration and social support, clinic directors and providers observed that heavy workloads had a detrimental effect on clinic staff morale. To motivate clinic staff, a clinic director successfully created a social group for clinic staff, where staff provided support and encouragement for each other. Similarly, clinic providers from a different clinic explained the importance of collaboration between staff in the MMT and HIV sections:

When the MMT section is too crowded, [the HIV provider] will help and vice versa when the HIV section needs assistance, [the MMT provider] will help… People keep working and assisting each other. When it’s too crowded, we all help each other and then get back to normal work. (Clinic Provider, Pilot Clinic 1)

Intra-clinic collaboration increased support and communication among staff and, at the same time, increased knowledge of respective MMT and HIV responsibilities.

#### Local commitment

Clinic directors from both integration pilot clinics expressed the need for strong and unified support from health and social service authorities, hospital directors, and hospital departments.

To create local champions for integration, a stakeholder at the central level recommended providing local leaders, such as clinic directors, with pilot evidence to generate buy-in. Once buy-in is established, leaders should be provided with technical assistance to facilitate the integration process.

I think that the key driver for success is the local leadership. If neither PAC [Provincial AIDS Center] nor the leaders recognize that important role, it is impossible to work out. The very first thing we did was to convince the leaders, to provide them with project evidence…Once they realize it, they lead, we just provide them technical support, advice on steps. If a challenge occurred, we further advise. We have the developed plan available to facilitate the process, to monitor it and we transfer the technology to them. (NGO)

Some stakeholders also mentioned local leadership commitment as a mechanism for obtaining investments for infrastructure, equipment, human resources, and policies to facilitate integration.

### Phase 2

#### Implementation strategies

Vietnamese stakeholders (*n* = 4) and implementation science experts (*n* = 5) scored each potential implementation strategy from 1 to 10 based on feasibility and importance, noting the rationale for their scores (Table 3).

**Table 3:** Summary of potential implementation strategies, median scores, and score ranges

Domain	Strategy	Median	Range
Technical assistance	External technical assistance and ongoing consultation	9	7–10
	Technical assistance within the clinic	8	7–10
	Educational outreach visit	7	5–9
	Ongoing and dynamic training	7.5	4–10
	Educational materials	6.5	4–10
Accountability	Audit and provide feedback	8	7–10
	Real-time relay of clinical data	8	7–10
	Quality monitoring tools	7.5	5–9
	Clinical implementation team meetings	7.5	5–9
	Clinical supervision	6.5	4–10
	Reminders to clinicians	6	5–10
Local commitment	Identify champions	9	5–9
	Capture local knowledge	8.5	7–10
	Build coalition	7.5	7–10
	Advisory boards/workgroups	6	4–10
	Executive boards	6	4–10

We provided a short description of the Vietnamese context and of each implementation strategy on the survey. In general and as anticipated, the Vietnamese stakeholders were not as familiar with specific implementation strategies whereas the implementation science experts were not as familiar with the Vietnamese context. This difference in familiarity affected scores on clinic-level strategies such as clinic reminders and relay of clinical data. Several of the strategies had a narrow range of scores, suggesting convergence of opinions across members of the expert panel.

### Technical assistance for clinic staff

All potential strategies within this domain were scored highly. The lowest median score was 6.5 (educational materials), the highest median score was 9 (external technical assistance and ongoing consultation), and scores ranged in total between 4 and 10.

Vietnamese stakeholders and implementation science experts provided similar responses with regard to implementation strategies in the area of technical assistance with the exception of educational materials and ongoing and dynamic training. For educational materials, the Vietnamese stakeholders had a median score of 9.5 and a range of between 7 and 10 while the implementation science experts had a median score of 6 and a range of between 4 and 7. One implementation science expert provided a score of 6, noting that educational materials are necessary but not effective for behavior change. This comment offers one explanation for why this strategy had a lower range among the implementation experts. For ongoing and dynamic training, both Vietnamese stakeholders and implementation science experts had a median score of 8. However, Vietnamese stakeholders had a narrower range (6–9) compared to implementation science experts (4–10).

Overall, implementation experts commented that they had a difficult time scoring the strategies within this domain because they felt that the most promising strategies had been identified and that all of these strategies were important and feasible. Vietnamese stakeholders also commented that the strategies within this domain were inherently interconnected.

### Staff accountability

The median scores within this domain were slightly lower than scores in the areas of technical assistance and local commitment. The lowest median score for a strategy was 6 (reminders to clinicians) while the highest median score for a strategy was 8 (audit and provide feedback, real-time relay of clinical data). Scores ranged between 4 and 10. The median scores of Vietnamese stakeholders and implementation science experts were similar. However, Vietnamese stakeholders had wide ranges (3–10) in their scoring of two strategies (audit and provide feedback, real-time relay of clinical data). These wide ranges were largely attributable to one stakeholder who commented that these proposed strategies were less feasible than others. Vietnamese stakeholders also had a narrow range (7–9) of scores for the strategy of clinical implementation team meetings, suggesting a high level of agreement on the importance and feasibility of this implementation strategy.

Implementation science experts had a wide range of scores (4–10) for the strategy of clinical supervision. However, no additional commentary was provided that would allow us to determine possible reasons for this range. Two implementation science experts did note that lack of context-specific knowledge hampered their ability to evaluate whether clinical reminders would be helpful because in the USA, providers often ignore reminders. This may explain this strategy’s median score of 6, the lowest within this domain.

### Local commitment

Similar to technical assistance, all potential strategies within this domain were scored highly with a lowest median score of 6 (executive boards), a highest median score of 9 (identify champions), and a total range of scores between 4 and 10.

Vietnamese stakeholders and implementation science experts scored most of the strategies within this domain similarly. However, there were differences in median scores for two of the strategies (build coalition, identify champions). Vietnamese stakeholders scored “build a coalition” higher (9.5) than the implementation science experts (7). On the other hand, implementation science experts scored “identify champions” higher (10) than the Vietnamese stakeholders (7.5), noting that local champions or opinion leaders are crucial to implementation.

The scores for the strategies within this domain varied widely (range of 4–9 or 4–10) among Vietnamese stakeholders. Some of this disparity is attributable to stakeholders who believed that strategies within this domain are less feasible than those from other domains.

### Recommended implementation strategies

Based on the results of this questionnaire and further discussions with our expert panel, we identified the following domains and strategies to be of primary importance for facilitating MMT and HIV service integration: (1) technical assistance including the strategies: centralize ongoing internal or external technical assistance; periodic educational outreach; and development of educational materials; (2) staff accountability including the strategies: create audit and feedback loops; provide clinical supervision; and clinical implementation team meetings;( 3) local commitment including the strategies: identify and prepare champion individual; build inter-clinic coalition where knowledge is shared; and involve an executive board.

## Discussion

Integration of MMT/HIV services is recognized as an EBI and is recommended by the US Centers for Disease Control and Prevention, the US Department of Health and Human Services, and the World Health Organization [53, 54]. Overall, integration benefits PWID through reduced bureaucratic and logistical barriers, increased monitoring of drug interactions, increased attention to stigma among providers, consolidated HIV risk and drug counseling, and targeted, user-friendly service delivery [46–48, 51]. In addition to patient benefits, integration also benefits healthcare systems by reducing costs and improving efficiencies in care [26, 49, 50], with economic models in Ukraine and Vietnam demonstrating that MMT/HIV integration is a more cost-effective intervention than ART alone [51, 52].

Co-locating staff is typically insufficient for ensuring collaboration or continuity of care [49]. Indeed, implementation strategies are needed to address barriers and build on facilitators to guarantee effective integration of MMT and HIV services. Given the strong evidence for integration and the lack of clarity on best practices for implementation in specific settings [4, 14], we identified potential implementation strategies for the Vietnamese context that align with the domains of technical assistance, staff accountability, and local commitment.

In Phase 1 of our study, participants agreed that integration has multiple benefits to both patients and the health care system and were generally open to MMT/HIV integration. However, barriers at multiple levels were noted. Barriers and facilitators to integration fell into two domains modifiable at the policy level, service and staffing structures, and three domains modifiable at the programmatic level, technical assistance for clinic staff, staff accountability, and local commitment. At the policy level, the need for a central-level decree was widely acknowledged and considered essential before clinics could begin the process of integration. In addition, differences in insurance reimbursements and staff salaries for MMT services as compared to HIV services need to be harmonized before these two service structures can integrate. Concerns about staffing reductions and resistance to taking on additional tasks without compensation were voiced by clinic-level staff and recognized by leadership, highlighting the need to develop clear policies for shifting staff and for providing monetary and non-monetary rewards for additional tasks. The VAAC is actively working on these two policy barriers.

At the programmatic level, clinic staff provided detailed feedback on what would be needed to operationalize clinic integration. Main challenges within clinics were lack of cross-training in HIV and MMT related tasks among clinic staff, lack of communication between MMT and HIV service providers, and lack of motivation to integrate effectively. Clinic stakeholders from pilot integration clinics reported that they were able to integrate MMT and HIV services effectively, illustrating that policy or programmatic barriers to integration are not insurmountable and that implementation strategies can address challenges to service integration. They observed that programmatic level facilitators, such as staff training, cross-clinic collaboration, real-time information sharing, and local leadership commitment, played a large role in their success.

The key implementation barriers identified in this study, including reluctance to add tasks to provider workloads, lack of staff training, and lack of commitment from local leaders, have been observed in other settings [50, 75–77], although specific combinations of barriers and solutions vary across country contexts. For example, in Ukraine, regulatory bodies at the central level did not permit nurses to simultaneously dispense TB and ART [75]. In contrast, in Tanzania, co-dispensing is easily accomplished without regulatory hurdles while the integration of MMT and HIV services is impeded by a shortage of trained providers [76]. Similar to Vietnam, China has faced inefficient coordination between MMT and HIV clinics driven by barriers including insufficient training and ongoing guidance, resistance to added workload, and PWID-related stigma among HIV providers [77]. Given these varied contexts and the challenges faced, a process for quickly determining barriers and tailored solutions is essential.

Based on the demonstrated need for technical assistance, staff accountability, and local commitment identified in Phase 1, we explored, in Phase 2, potential implementation strategies aligned with these domains and, through a voting process, selected the most promising implementation strategies. Specifically, we used a compilation of strategies [16] and consulted with implementation experts to select a total of 16 potential strategies for an importance and feasibility poll.

Inclusion of both Vietnamese stakeholders and implementation science experts in the voting panel ensured expertise in both the organizational and cultural context of Vietnam as well as the effectiveness of strategies for implementing a range of EBIs. The number of members in the panel (*n* = 9) allowed for both a range of perspectives as well as a process that was not overly burdensome in terms of following up with members and managing qualitative responses.

As expected, scoring by Vietnamese stakeholders and implementation scientists tended to cluster, especially in certain domains. For example, Vietnamese stakeholders had a median score of 9 for educational materials under technical assistance, while implementation science experts had a median score of 6. The higher score awarded by the Vietnamese experts reflects the familiarity, feasibility, and acceptability of educational materials in the Vietnamese setting, while the lower score, given by implementation experts, stemmed from experience in the USA where educational materials are not independently effective in changing behaviors.

Combining quantitative and qualitative findings to inform our strategy selection process was particularly helpful given the small size of our voting expert panel. Specifically, rather than simply selecting 3–4 strategies with the highest ranking scores, we also considered the qualitative factors that influenced scores (elicited from both the written comments, and sometimes, verbal or email follow-up for further clarification as needed) as part of our decision-making process. As a result, although educational materials only had a median of 6.5, we included it in the technical assistance package since Vietnamese stakeholders felt it was highly acceptable and feasible and the implementation science experts believed it was a necessary strategy within the technical assistance domain.

## Conclusions

Our study applied an adapted ERIC protocol [14] over a 3-month period to support a streamlined process for identifying culturally salient and context-appropriate EBI implementation strategies. Our process consisted of four steps within two main phases. In Phase 1, we conducted 16 in-depth interviews with particular insight into integration in Vietnam and developed matrices to display barriers and facilitators. In Phase 2, we selected implementation strategies that addressed the barriers identified in Phase 1 and conducted a poll to vote on the most important and feasible strategies with an expert panel that incorporated expertise in cultural context and implementation science. This process was relatively simple, low cost, and potentially replicable in other settings.

Our stakeholders represented three levels of the Vietnamese health system (central-level policy makers, Department of Health and clinic directors, and clinic staff), three provinces, and both stand-alone and integrated clinics. While our sample sizes were relatively small, they provided us with the necessary breadth and depth to address MMT/HIV integration in Vietnam. Qualitative data enhanced the utility of our importance and feasibility poll by providing rich insight into dimensions of each implementation strategy that may have influenced the selection of certain scores. The process of selecting our final strategies relied on a combination of poll results and qualitative dialogue. Our study sheds light on barriers and facilitators to MMT/HIV integration, which may be generalizable to other low-income settings. Furthermore, our process produced promising implementation strategies to facilitate MMT/HIV service integration, addressing gaps in both the substance use and implementation science literature. These implementation strategies must be further evaluated in a field trial, using both effectiveness and implementation outcomes. If these implementation strategies are effective, next steps would include determining whether these implementation strategies can be adapted to other settings and expanded to other comorbid conditions among PWID.

## Additional files

Additional file 1: Stakeholder interview guide: VAAC. (DOCX 134 kb)

Additional file 2: Stakeholder interview guide: pilot-integrated clinic staff. (DOCX 130 kb)

Additional file 3: Stakeholder interview guide: FHI and USAID. (DOCX 125 kb)

Additional file 4: Stakeholder interview guide: non-pilot director provincial Department of Health. (DOCX 18 kb)

Additional file 5: Stakeholder interview guide: pilot director provincial Department of Health. (DOCX 18 kb)

## Abbreviations

ART: antiretroviral therapy
ARV: antiretroviral
EBI: evidence-based intervention
ERIC: Expert Recommendations for Implementing Change
IDI: in-depth interview
MMT: methadone maintenance therapy
NGO: non-governmental organization
PAC: Provincial AIDS Center
PWID: people who inject drugs
UNC: University of North Carolina at Chapel Hill
VAAC: Vietnam Administration for HIV/AIDS Control
