# Supplementary material for: Finding what works: identification of implementation strategies for the integration of methadone maintenance therapy and HIV services in Vietnam
Source: Implement Sci. 2016 Apr 20;11:54. doi: 10.1186/s13012-016-0420-8 (PMC4837557; doi:10.1186/s13012-016-0420-8)
Supplement: Supplementary file 1 — Stakeholder interview guide: VAAC. (DOCX 134 kb) [file 13012_2016_420_MOESM1_ESM.docx]

**Stakeholder Interview Guide: VAAC (n = 2)**

**Objectives:** *To identify national, provincial and clinic level barriers and facilitators to the integration of HIV and MMT services in Vietnam and to explore possible recommendations for overcoming these challenges.*

**Introduction**

1. Please tell me a little about your role at the Vietnam Administration for AIDS Control.
   1. Probe: How long have you worked here?
   2. Probe: What are your responsibilities here?
2. What are some of the challenges you face in your role?
   1. Hint: limited staff, limited funds, bureaucracy, competing demands by international organizations.

**Definition of integration of HIV/MMT services**

*Today we’ll be talking about integration of HIV/MMT services. When we say integrated HIV/MMT clinics, we mean outpatient clinics that offer both methadone maintenance therapy and HIV services including HIV testing & ART. Integration can take many forms including staff that delivers both HIV and methadone services: for example, having the same pharmacist at the clinic dispensing both ART and methadone. It could also mean having integrated space: for example, having a secure storeroom that stores both ART and other HIV medications and methadone.*

*I’d like to talk to you about how you envision integration of services, what you think the pros and the cons of integration are, and what you think are some barriers and facilitators to integrating services. Let’s begin by talking about definitions of HIV/MMT services.*

1. There are many different ways to integrate HIV and MMT services. To your knowledge, how does VAAC define the integration of HIV/MMT services:
   1. Probe: co-location of services, offering both services at the same clinic, offering both services by the same staff, integrated database/paperwork requirements.
   2. Probe: What are some differences that the VAAC could expect across provinces and clinic in terms of implementing integration of services?
2. Currently, is integrating HIV/MMT services a national goal in Vietnam? How does it rate compared to other national goals? Does VAAC have a plan or timeline for integration of HIV/MMT services
   1. Probe: is there a date for implementation of integration of services? What else needs to be in place in order for integration to occur?
3. From what you know, to make HIV/MMT services a national set of goals, which national and international agencies/organizations/people would need to support this policy? (hint: MOH, Life Gap, Global Fund, VAAC)
   1. Probe: Of the organizations you named, who supports integration of services and what do you think their rationale is?
   2. Probe: Of the organizations you named, who does not actively support integration or are against integration of services, and why do you think they do not support these services?

**Pros and cons of integration of HIV/MMT services.**

1. What do you think about the integration of HIV/MMT services as a part of a national goal?
   1. Probe: What do you think are main advantages (hint: saves resources, facilitates access to IDU).
   2. Probe: what do you think are the main disadvantages (hint: restructuring, re-training, turf wards).

**Barriers and facilitators to integration of services**

1. Let’s say that you wanted to initiate a national goal to integrate HIV and MMT services in Vietnam for the VAAC. Walk me through what exactly would need to be done in order to integrate services from the national level, down to the clinic level.
   1. Probe: What are main steps that would need to be accomplished at the national level?
   2. Probe: What would need to be done at the provincial level?
   3. Probe: What steps would need to be taken by the clinics?
2. In your opinion, what are some of the main challenges to integration of HIV/MMT services?
   1. Hint: training, buy –in from key agencies at the national level, buy-in from staff and clinic directors—why?
3. There have been some pilot integrated clinics in Hai Phong and in other areas. How many pilot integrated clinics are in the country. In your opinion, how successfully did those clinics integrate?
4. To your knowledge, what were the barriers related to integration of services at these pilot clinics?
   1. Probe: What are some ways that could overcome these barriers?
   2. Probe: Do you know of any strategies or approaches that helped to make these clinics work?
5. If you had a chance to oversee integration again, what would you do differently? How would you have changed the steps taken by VAAC to integrate services in clinics?
6. Is there anyone else that you recommend that I talk to about the integration of services?
