# Supplementary material for: Finding what works: identification of implementation strategies for the integration of methadone maintenance therapy and HIV services in Vietnam
Source: Implement Sci. 2016 Apr 20;11:54. doi: 10.1186/s13012-016-0420-8 (PMC4837557; doi:10.1186/s13012-016-0420-8)
Supplement: Supplementary file 2 — Stakeholder interview guide: pilot-integrated clinic staff. (DOCX 130 kb) [file 13012_2016_420_MOESM2_ESM.docx]

**Stakeholder Interview Guide: Pilot Integrated Clinic Staff**

**(n = 9: 2 in pilot integrated clinic, 2 in MMT clinics, 2 in HIV clinics; 1 clinic director of pilot clinic; 1 HIV clinic director 1 MMT clinic director )**

Interviewer note: This guide is for two types of clinic staff— those who are working in a pilot integrated clinic and those who are working in stand alone MMT/HIV clinics. Text that is underlined and in [brackets] is for the staff who are in stand alone MMT/HIV clinics.

**Objectives:** *To explore the perceptions, attitudes, barriers and facilitators to the integration of HIV and MMT services and Vietnam and to explore possible recommendations for overcoming these challenges.*

**Introduction**

1. Please tell me a little about your role at this clinic.
   1. Probe: How long have you worked here?
   2. Probe: What are your responsibilities here?
2. What are some of the challenges you face in your role?
   1. Hint: limited staff, overworked, limited funds.
3. Can you tell me how this clinic is staffed?
   1. Probe: how many physicians, nurses, receptionists, counselors
4. Can you tell me about your patient population?
   1. Probe: on a typical day, how many patients does the clinic see daily?
   2. Probe: on a typical day, how many patients do you serve?
   3. Probe: Are there patterns in patient flow? Is there more patient traffic during certain times of the day?
   4. Probe: what is the ratio of MMT to HIV patients? About what proportion get both types of services, MMT alone, HIV alone. *[stand alone HIV/MMT clinic staff, please skip this question]*

**Perceptions of integrating HIV/MMT services**

*We’d like to talk to you a little more about your experiences with the integration HIV/MMT services in your province so that we can learn from your experiences in integrating services and plan for future integrated clinics. When we say integrated HIV/MMT clinics, we mean outpatient clinics that offer both methadone maintenance therapy and HIV services including HIV testing & ART. Integration can take many forms including staff that delivers both HIV and methadone services: for example, having the same pharmacist at the clinic dispensing both ART and methadone. It could also mean having integrated space: for example, having a secure storeroom that stores both ART and other HIV medications and methadone.*

1. What are the advantages and disadvantages of integration of HIV/MMT services?
   1. Probe: What do you think are main advantages (hint: saves resources, facilitates access to IDU).
   2. Probe: what do you think are the main disadvantages (hint: restructuring, re-training, turf wards).

**Process, barriers and facilitators to integration of services**

1. Has your clinic ever tried to offer both HIV and MMT services? If *yes*, can you tell me how you think the services were integrated? *[If no, please skip this question]*
   1. Probe: Prior to integration, was the clinic an existing ART or MMT clinic, or was it a new clinic?
   2. What were the steps that the clinic took in order to integrate HIV/MMT services into one clinic? Walk me through what exactly was done in your clinic to integrate services?
   3. hint: training, spaces reallocation, meetings with different staff members, how long it took.
2. Can you tell me how (you think) integration of services [would] affected you and your role?
   1. Hint: change in work load: more work, less work, the same
   2. Probe: how do you feel about the change in your work responsibilities? (hint: interesting, not interesting, neutral). [How would you feel about being trained in HIV/MMT care? How would you feel about delivering services to people living with drugs/injecting drug users?]
3. To your knowledge, how did [would] other staff members feel about the integration of HIV/MMT services?
   1. Probe: pharmacists, physicians, receptionists, counselors, nurses.
4. In your opinion, what are some of the main challenges to integrating HIV/MMT services?
   1. Hint: training, buy –in from key agencies at the national level, buy-in from staff and clinic directors—why?

*Questions 5-7 for pilot clinic staff only*

1. In your opinion, how successfully did your clinic integrate?
   1. Probe: can you tell me a little about clinic attendance? (decreased, stayed the same, increased)?
   2. Probe: can you tell me about patient satisfaction with services?
2. To you knowledge, what were the barriers related to integration of services at your clinic?
   1. Probe: What are some ways that could overcome these barriers?
   2. Probe: Do you know of any strategies or approaches that helped to make these clinics work?
3. If you had a chance to do this again, what would you do differently? How would you have changed the way that services were integrated in your clinic?
   1. Hint: more trainings, more space, more money.

*[Questions 8-9 for non pilot clinic staff only]*

1. In your opinion, how difficult do you think it would be to offer HIV and MMT services at your clinic?
   1. What would make it difficult?
   2. Is there anything that would make it easier/smoother?
2. If there was a plan to integrate services in your clinic, what would you recommend doing to make this transition work well for the staff in your clinic?
   1. Hint: training, on-call expertise, integrate database.
