# Supplementary material for: Finding what works: identification of implementation strategies for the integration of methadone maintenance therapy and HIV services in Vietnam
Source: Implement Sci. 2016 Apr 20;11:54. doi: 10.1186/s13012-016-0420-8 (PMC4837557; doi:10.1186/s13012-016-0420-8)
Supplement: Supplementary file 3 — Stakeholder interview guide: FHI and USAID. (DOCX 125 kb) [file 13012_2016_420_MOESM3_ESM.docx]

**Stakeholder Interview Guide: FHI and USAID (pilot implementing agencies) (n = 1)**

**Objectives:** *To identify lessons learned from pilot HIV/MMT integrated clinics in Vietnam. Specifically we would like to understand barriers and facilitators to integration of services and to identify possible strategies for overcoming major challenges.*

**Introduction**

1. Please tell me about your role at FHI/USAID.
   1. Probe: how long have your worked here in this position?
   2. What are your responsibilities here?

**Definition of Integration of HIV/MMT services**

*Today, we’ll be talking about the integration of HIV/MMT services. We’re particularly interested in learning about your experiences from your pilot clinics.*

1. There are many different ways to integrate HIV and MMT services. Can you tell me how you would define the integration of HIV and MMT services?
2. Can you tell me a little about the pilot clinics that integrated HIV/MMT services?
   1. Probe: What date did the pilot clinic begin to operate as an integrated clinic?
   2. Probe: Are they currently operating as an integrated clinic?
   3. Probe: How many pilot clinics were there, and where were they located?
   4. Probe: Which agency (s) sponsored these pilots?
3. How are HIV and MMT services operationalized in the pilot clinics?
   1. Probe: are services located in one clinic?
   2. Probe: Was it a pre-existing ART or MMT clinic or was it a new clinic?
   3. Probe: which staff roles/responsibilities are integrated: receptionist, pharmacists, counseling, physician?
   4. Probe: how is the paperwork/databases managed? Were they integrated?
   5. Probe: how is physical layout integrated between the services? Were the spaces separated by service, or were some spaces shared between services?

**Perceptions of integration of HIV/MMT services**

1. Can you tell me a little about the different Vietnamese and international organizations involved in the pilot integration.
   1. Probe: which organizations were involved and what were their roles? International/national, provincial level, sub-district level
   2. Probe: were certain organizations resistant or hesitant to integrate services? Why do you think they were resistant?
   3. Probe: did certain organizations actively support integration? Why do you think they were supportive?
2. Can you tell me about the different types of people that were involved in implementing the pilot clinic and their roles?
   1. Probe: VAAC director and leadership, director provincial health services, director of provincial AIDS committee, harm reduction director, clinic director, clinic staff.
   2. Probe: How did each of these people feel about integration? Were they supportive, resistant, neutral, why?
3. From what you know, to make HIV/MMT services a national policy, which national and international agencies/organizations/people would need to support this policy? (hint: MOH, Life Gap, Global Fund, VAAC)
   1. Probe: Of the organizations you named, who supports integration of services and what do you think their rationale is?
   2. Probe: Of the organizations you named, who does not actively support integration or are against integration of services, and why do you think they do not support these services?

**Pros and cons of integration of HIV/MMT services.**

1. What do you think about the integration of HIV/MMT services as a national policy?
   1. Probe: What do you think are main advantages (hint: saves resources, facilitates access to IDU).
   2. Probe: what do you think are the main disadvantages (hint: restructuring, re-training, turf wards).

**Barriers and facilitators to integration of services**

1. In your opinion, how successful do you think the pilot clinics were at providing integrated services?
2. Based on the pilot clinics, what are some of the main challenges to integration of HIV/MMT services?
   1. Hint: training, buy –in from key agencies at the national level, buy-in from staff and clinic directors—why?
3. What are some strategies or approaches that could overcome these barriers?
   1. Probe: Do you know of any strategies or approaches that helped to make these clinics work?
4. Is there anyone else that you recommend that I talk to you about the integration of services?
