# Supplementary material for: Finding what works: identification of implementation strategies for the integration of methadone maintenance therapy and HIV services in Vietnam
Source: Implement Sci. 2016 Apr 20;11:54. doi: 10.1186/s13012-016-0420-8 (PMC4837557; doi:10.1186/s13012-016-0420-8)
Supplement: Supplementary file 4 — Stakeholder interview guide: non-pilot director provincial Department of Health. (DOCX 18 kb) [file 13012_2016_420_MOESM4_ESM.docx]

**Stakeholder Interview Guide: Non-Pilot Director Provincial Department of Health**

**(n = 1)**

**Objectives:** *To explore the perceptions, attitudes, barriers and facilitators to the integration of HIV and MMT services and Vietnam and to explore possible recommendations for overcoming these challenges.*

Interviewer note: This guide is for a Director who does not have a pilot integrated clinic in his province.

**Introduction**

1. Please tell me a little about your role at provincial health services.
   1. Probe: How long have you worked here?
   2. Probe: What are your responsibilities here?
2. What are some of the challenges you face in your role?
   1. Hint: limited staff, limited funds, bureaucracy, competing demands by international organizations.
3. How many ART clinics are there in the province? How many MMT clinics are there in the province? How are these clinics staffed?
   1. Hint: number of physicians, pharmacists, counselors, nurses

**Perceptions of integrating HIV/MMT services**

*We’d like to talk to you a little more about your experiences with the integration HIV/MMT services in Vietnam. When we say integrated HIV/MMT clinics, we mean outpatient clinics that offer both methadone maintenance therapy and HIV services including HIV testing & ART. Integration can take many forms including staff that delivers both HIV and methadone services: for example, having the same pharmacist at the clinic dispensing both ART and methadone. It could also mean having integrated space: for example, having a secure storeroom that stores both ART and other HIV medications and methadone. We’d like to hear about your thoughts on integration of services.*

1. Can you tell me about how you would integrate HIV and MMT services?
   1. Probe: How would you integrate services into one clinic?
      1. Hint: Would you use an existing ART or MMT clinic or create a new clinic?
   2. Probe: How would you staff the integrated clinic?
      1. Hint: number of physicians, pharmacists, counselors, nurses
      2. Hint: where would the staff come from—existing MMT and HIV clinics, newly hired?
2. What do you think about the integration of HIV/MMT services?
   1. Probe: What do you think are the main advantages (hint: saves resources, facilitates access to IDU).
   2. Probe: What do you think are the main disadvantages (hint: restructuring, re-training, turf wards).

**Barriers and facilitators to integration of services**

1. What are the steps that you would have to take to take in order to integrate HIV/MMT services into one clinic? Walk me through what exactly would need to be done in order to integrate services from the provincial level, down to the clinic level.
   1. Probe: What would need to be done at the provincial level?
   2. Probe: What steps would need to be taken by the clinics? (hint: training, spaces reallocation, meetings).
2. In your opinion, what are some of the main challenges to integration of HIV/MMT services?
   1. Hint: training, buy –in from key agencies at the national level, buy-in from staff and clinic directors—why?
3. In your opinion, how successfully would these clinics integrate?
4. To you knowledge, what would be the barriers related to integration of services at these integrated clinics?
   1. Probe: What are some ways that could overcome these barriers?
   2. Probe: Do you know of any strategies or approaches that helped to make these clinics work?
