# Supplementary material for: Finding what works: identification of implementation strategies for the integration of methadone maintenance therapy and HIV services in Vietnam
Source: Implement Sci. 2016 Apr 20;11:54. doi: 10.1186/s13012-016-0420-8 (PMC4837557; doi:10.1186/s13012-016-0420-8)
Supplement: Supplementary file 5 — Stakeholder interview guide: pilot director provincial Department of Health. (DOCX 18 kb) [file 13012_2016_420_MOESM5_ESM.docx]

**Stakeholder Interview Guide: Pilot Director Provincial Department of Health (n = 1)**

**Objectives:** *To explore the perceptions, attitudes, barriers and facilitators to the integration of HIV and MMT services and Vietnam and to explore possible recommendations for overcoming these challenges.*

Interviewer note: This guide is for a Director who has a pilot integrated clinic in his province.

**Introduction**

1. Please tell me a little about your role at provincial health services.
   1. Probe: How long have you worked here?
   2. Probe: What are your responsibilities here?
2. What are some of the challenges you face in your role?
   1. Hint: limited staff, limited funds, bureaucracy, competing demands by international organizations.
3. How many ART clinics are there in the province? How many MMT clinics are there in the province? How are these clinics staffed?
   1. Hint: number of physicians, pharmacists, counselors, nurses

**Perceptions of integrating HIV/MMT services**

*We’d like to talk to you a little more about your experiences with the integration HIV/MMT services in Vietnam. When we say integrated HIV/MMT clinics, we mean outpatient clinics that offer both methadone maintenance therapy and HIV services including HIV testing & ART. Integration can take many forms including staff that delivers both HIV and methadone services: for example, having the same pharmacist at the clinic dispensing both ART and methadone. It could also mean having integrated space: for example, having a secure storeroom that stores both ART and other HIV medications and methadone. We’d like to learn from your experiences in integrating services so that we can plan for future integration of services.*

1. Can you tell me a little about the integrated clinic?
   1. Probe: How were services integrated into one clinic?
      1. Hint: Prior to integration, was the clinic an existing ART or MMT clinic, or was it a new clinic?
   2. Probe: How was the integrated clinic staffed?
      1. Hint: number of physicians, pharmacists, counselors, nurses
      2. Hint: where did the staff come from—existing MMT and HIV clinics, newly hired?
2. What do you think about the integration of HIV/MMT services?
   1. Probe: What do you think are the main advantages (hint: saves resources, facilitates access to IDU).
   2. Probe: What do you think are the main disadvantages (hint: restructuring, re-training, turf wards).

**Barriers and facilitators to integration of services**

1. What were the steps that you had to take in order to integrate HIV/MMT services into one clinic? Walk me through what exactly would need to be done in order to integrate services from the provincial level, down to the clinic level.
   1. Probe: What would need to be done at the provincial level?
   2. Probe: What steps would need to be taken by the clinics? (hint: training, spaces reallocation, meetings).
2. In your opinion, what are some of the main challenges to integration of HIV/MMT services?
   1. Hint: training, buy –in from key agencies at the national level, buy-in from staff and clinic directors—why?
3. In your opinion, how successfully did those clinics integrate?
4. To you knowledge, what were the barriers related to integration of services at these integrated clinics?
   1. Probe: What are some ways that could overcome these barriers?
   2. Probe: Do you know of any strategies or approaches that helped to make these clinics work?
